# Treatment of chronic hepatitis B in sub-Saharan Africa: 1-year results of a pilot program in Ethiopia

**DOI:** 10.1186/s12916-018-1229-x

**Published:** 2018-12-17

**Authors:** Hailemichael Desalegn, Hanna Aberra, Nega Berhe, Bitsatab Mekasha, Kathrine Stene-Johansen, Henrik Krarup, Andre Puntervold Pereira, Svein Gunnar Gundersen, Asgeir Johannessen

**Affiliations:** 1 Medical Department, St. Paul’s Hospital Millennium Medical College, Addis Ababa, Ethiopia; 2 Aklilu Lemma Institute of Pathobiology, Addis Ababa University, Addis Ababa, Ethiopia; 3 Centre for Imported and Tropical Diseases, Oslo University Hospital, Ullevål, PO Box 4956 Nydalen, 0424 Oslo, Norway; 4 Department of Molecular Biology, Norwegian Institute of Public Health, Oslo, Norway; 5 Section of Molecular Diagnostics, Aalborg University Hospital, Aalborg, Denmark; 6 Faculty of Medicine, University of Oslo, Oslo, Norway; 7 Research Unit, Sørlandet Hospital HF, Kristiansand, Norway; 8 Department of Global Development and Planning, University of Agder, Kristiansand, Norway; 9 Department of Infectious Diseases, Vestfold Hospital Trust, Tønsberg, Norway

**Keywords:** Viral hepatitis, Antiviral therapy, Resource-limited settings, Epidemiology

## Abstract

**Background:**

The World Health Organization has set an ambitious goal of eliminating viral hepatitis as a major public health threat by 2030. However, in sub-Saharan Africa, antiviral treatment of chronic hepatitis B (CHB) is virtually unavailable. Herein, we present the 1-year results of a pilot CHB treatment program in Ethiopia.

**Methods:**

At a public hospital in Addis Ababa, CHB patients were treated with tenofovir disoproxil fumarate based on simplified eligibility criteria. Baseline assessment included liver function tests, viral markers, and transient elastography (Fibroscan). Changes in laboratory markers were analyzed using Wilcoxon signed-rank tests. Adherence to therapy was measured by pharmacy refill data.

**Results:**

Out of 1303 patients, 328 (25.2%) fulfilled the treatment criteria and 254 (19.5%) had started tenofovir disoproxil fumarate therapy prior to September 1, 2016. Of the patients who started therapy, 30 (11.8%) died within the first year of follow-up (28 of whom had decompensated cirrhosis), 9 (3.5%) self-stopped treatment, 7 (2.8%) were lost to follow-up, and 4 (1.6%) were transferred out. In patients who completed 12 months of treatment, the median Fibroscan value declined from 12.8 to 10.4 kPa (*p* < 0.001), 172 of 202 (85.1%) patients with available pharmacy refill data had taken ≥ 95% of their tablets, and 161 of 189 (85.2%) patients with viral load results had suppressed viremia. Virologic failure (≥ 69 IU/mL) at 12 months was associated with high baseline HBV viral load (> 1,000,000 IU/mL; adjusted OR 2.41; 95% CI 1.04–5.55) and suboptimal adherence (< 95%; adjusted OR 3.43, 95% CI 1.33–8.88).

**Conclusions:**

This pilot program demonstrated that antiviral therapy of CHB can be realized in Ethiopia with good clinical and virologic response. Early mortality was high in patients with decompensated cirrhosis, underscoring the need for earlier detection of hepatitis B virus infection and timely initiation of treatment, prior to the development of irreversible complications, in sub-Saharan Africa.

**Trial registration:**

NCT02344498 (ClinicalTrials.gov identifier). Registered 16 January 2015.

## Background

Chronic infection with hepatitis B virus (HBV) is a leading cause of cirrhosis, hepatic decompensation, and hepatocellular carcinoma (HCC). Approximately 257 million people worldwide are living with chronic hepatitis B (CHB), and an estimated 887,000 deaths per year are attributable to HBV infection [1]. Despite an effective vaccine and potent antiviral drugs, the number of HBV-related deaths has increased by 33% between 1990 and 2013 [2]. Indeed, in 2015, viral hepatitis claimed more lives than human immunodeficiency virus (HIV) [3].

Studies from high-income countries have shown that antiviral treatment of CHB reduces the risk of disease progression [4]. Long-term antiviral treatment has been shown to stop and even reverse liver fibrosis and prevent development of HCC [5, 6]. Hepatitis B surface antigen (HBsAg) loss is the optimal treatment endpoint, but is rarely achieved; thus, most patients need long-term treatment to suppress viral replication and prevent hepatic complications [7].

In sub-Saharan Africa, treatment for viral hepatitis is rarely available in the public sector. Patients with CHB are left untreated and physicians are left to merely follow the natural course of the disease and provide palliative care. Paradoxically, the recommended first-line drug for CHB, tenofovir disoproxil fumarate (TDF), is registered in most African countries for the treatment of HIV, but not for hepatitis. Therefore, patients mono-infected with HBV cannot access life-saving treatment, whereas those with HIV/HBV co-infection receive free treatment through donor agencies.

In 2016, the World Health Organization (WHO) published the Global Health Sector Strategy of Viral Hepatitis [3] and set an ambitious goal of eliminating viral hepatitis as a public health threat by 2030. To meet the WHO goals, countries and regions should reduce new infection by 90% and deaths by 65% by 2030 [3, 8]. However, to date, few African countries have developed national action plans for viral hepatitis, and only one published study – the PROLIFICA study in The Gambia [9] – has reported results of HBV treatment on the continent. Consequently, there is a lack of local data to direct guidelines and promote implementation.

Herein, we present results from one of the first and largest public treatment programs for hepatitis B in sub-Saharan Africa. Many of the barriers to treatment in Ethiopia, such as lack of diagnostic facilities, absence of public funding, and restrictions on antiviral drugs, are shared by most low-income countries; therefore, we believe that our findings can be relevant in the global scaling-up of antiviral treatment of CHB.

## Methods

### Study setting and participants

Ethiopia is a low-income country in East Africa with an estimated prevalence of hepatitis B at 7.4% [10]. A pilot program for the treatment of CHB was established in February 2015 at St. Paul’s Hospital Millennium Medical College, which is a referral hospital in the capital Addis Ababa. A simplified approach to treatment and care of individuals with CHB was employed, wherein transient elastography rather than liver biopsy was used to assess liver fibrosis, nurses rather than physicians were responsible for most of the patient consultations, and treatment eligibility criteria were straight-forward and easy to use.

As this was the first public healthcare facility offering CHB treatment in the country, patients were referred from various hospitals, health centers, blood banks, and antenatal clinics for evaluation and treatment. Patients aged 18 years or above with CHB, defined as a persistently positive HBsAg test for more than 6 months, were enrolled in the program. Since previous HBsAg testing was usually performed in other hospitals or private clinics, and often years back, we accepted the patients’ recollection of a previous positive result without demanding a written laboratory report. Patients who were HIV positive at presentation were not included, but rather transferred to the nearest HIV care and treatment center. Likewise, individuals with a known terminal disease such as HCC were referred for further management elsewhere.

### Patient assessment and laboratory tests

At enrollment, all patients were interviewed by a trained nurse in their own language. A full diagnostic work-up was performed, including laboratory tests and transient elastography. The following laboratory tests were performed at the first visit:

- Point-of-care rapid diagnostic tests: HBsAg, HIV (further tests were not performed in patients found to be HBsAg negative or HIV positive)
- Routine chemistry: complete blood count, bilirubin, alanine aminotransferase (ALT), aspartate aminotransferase (AST), creatinine
- Serology: HBsAg, hepatitis C virus (HCV) antibody, hepatitis D virus (HDV) antibody
- HBV DNA viral load

Patients with signs or symptoms of decompensated liver disease, such as ascites or jaundice, were scheduled to see a physician within 1–2 weeks. These patients would usually receive symptom-directed therapy such as diuretics for ascites/edema; however, within our simplified setup, we did not systematically perform endoscopic treatment of esophageal varices or other more advanced procedures.

Patients without signs or symptoms of advanced liver disease were appointed to a physician after 3 months when the viral load result would usually be available. The decision to start therapy was made by a physician using the predefined criteria given below; otherwise, the follow-up was nurse led.

Those who started antiviral therapy were followed-up after 2 and 4 weeks, and thereafter 3-monthly. The main focus at each visit was adherence counseling (including pill count) and monitoring for side effects. Untreated individuals were followed-up at 3-month intervals.

The following laboratory tests were performed during follow-up (tests in parenthesis were only performed in patients on treatment):

- 3-monthly: complete blood count, ALT, AST, (creatinine, HIV rapid test)
- 6-monthly: HBsAg, HBV viral load

Blood tests were performed using commercially available kits and assays. HBsAg was detected on-site using a WHO-approved rapid diagnostic test (Determine, Alere Inc., USA). HIV testing was done in accordance with the National algorithm, i.e., using a WHO-approved rapid test kit (HIV 1+2 Antibody Colloidal Gold [KHB], Shanghai Kehua Bio-engineering co., China) for screening, and another rapid test kit (HIV 1/2 STAT-PAK, Chembio Diagnostics, USA) for confirmation. Other routine laboratory investigations for hematology (HumaCount 30, Human, Germany), biochemistry (Humalyzer 3000, Human, Germany), and serology (Elisys Uno, Human, Germany) were performed locally.

Aspartate aminotransferase to platelet ratio index (APRI) and FIB-4 were derived from standard blood test results using the following formulas:

$$ \mathrm{APRI}:\left(\mathrm{AST}\ \left[\mathrm{U}/\mathrm{L}\right]/\mathrm{upper}\ \mathrm{limit}\ \mathrm{of}\ \mathrm{normal}\ \mathrm{for}\ \mathrm{AST}\right)/\mathrm{platelet}\ \mathrm{count}\ \left({10}^9/\mathrm{L}\right)\times 100 $$

$$ \mathrm{FIB}-4:\left(\mathrm{age}\ \left[\mathrm{years}\right]\times \mathrm{AST}\ \left[\mathrm{U}/\mathrm{L}\right]\right)/\left(\mathrm{platelet}\ \mathrm{count}\ \left[{10}^9/\mathrm{L}\right]\times {\left(\mathrm{ALT}\ \left[\mathrm{U}/\mathrm{L}\right]\right)}^{1/2}\right) $$

HBV viral load testing was unavailable in Ethiopia at the time the program was set up; thus, baseline viral load testing was performed after shipment of samples to the Norwegian Public Health Institute (Oslo, Norway). The Abbott RealTime HBV assay (Abbott Molecular, Des Moines, USA) was used, following the manufacturer’s instructions. From 2016, HBV viral load testing was established at a private laboratory in Addis Ababa using the Abbott RealTime HBV assay and therefore all follow-up samples for HBV viral load monitoring were tested locally.

HDV antibodies were detected using an enzyme-linked immunosorbent assay (ELISA) method (ETI-AB-DELTAK-2, Diasorin, Italy) from EDTA plasma samples. A second anti-HDV ELISA assay (Dia.Pro Diagnostic Bioprobes Srl, Milan, Italy) was used to confirm indeterminate or weak positive results obtained with the Diasorin assay, as suggested by the manufacturer when plasma is used instead of serum. These analyses were performed at the Centre national de référence des hépatites B, C et Delta, Hôpitaux universitaires de Paris-Seine-Saint-Denis, France.

### Liver fibrosis assessment

Liver fibrosis was assessed using transient elastography (Fibroscan 402, Echosense, France). Patients were instructed to fast for at least 2 h prior to the examination, and the procedure was performed by an experienced operator as per the manufacturer’s instructions. The median of 10 readings was employed, and the result was discarded if the interquartile range (IQR) divided by the median exceeded 30%.

Based on a previous meta-analysis and a study from West Africa [11, 12], we used a Fibroscan threshold of 7.9 kPa to define significant fibrosis (corresponding to Metavir score ≥ F2) and 9.9 kPa to define cirrhosis (corresponding to Metavir score F4). Ultrasound of the liver was performed at baseline, and thereafter annually, in all patients who started treatment, mainly to detect HCC.

### Treatment eligibility

Since this program opened prior to the launch of the WHO Hepatitis B Guidelines in 2015 [13], treatment eligibility criteria were based on the European Association for the Study of the Liver (EASL) Guidelines from 2012 [7], with some modifications. Specifically, since liver biopsy was unrealistic in this setting, the two EASL criteria pertaining to liver inflammation (Metavir ≥ A2 with viral load > 2000 IU/mL and ALT > 80 U/L with viral load > 20,000 IU/mL) were merged into one, namely ALT > 80 U/L with viral load > 2000 IU/mL. Furthermore, since African patients with CHB are at particular risk of HCC, we created a new criterion for patients with HCC in their close family.

Thus, patients who fulfilled the following criteria were considered eligible for treatment:

1. Decompensated cirrhosis
2. Compensated cirrhosis (confirmed with ultrasound and/or Fibroscan)
3. Significant fibrosis (Fibroscan > 7.9 kPa) and viral load > 2000 IU/mL
4. Moderate/severe liver inflammation (ALT > 80 U/L) and viral load > 2000 IU/mL
5. HCC among first-degree relatives and viral load > 2000 IU/mL

Patients who met the treatment criteria were given adherence counseling and educated about the disease and the need for life-long follow-up. Preventive measures were recommended, including HBV vaccination to the patients’ partner and children. Based on its potency, safety profile, and high barrier to resistance, TDF (Viread, Gilead Sciences, lnc., Foster City, CA, USA) 300 mg once daily was used in this program. Treatment for HCV and HDV co-infections were not available through this program.

### Assessment of adherence to therapy

In patients who started antiviral treatment, TDF was initially dispensed for 1 month’s duration, and thereafter at 3-monthly intervals. At each visit to the clinic, the patients were told to bring their old pill boxes so that the remaining pills could be counted. Adherence was calculated by dividing the total amount of tablets dispensed by the total number of days since initiating therapy, expressed as percentage. This method (‘pharmacy refill’ or ‘pill count’) has previously been proven accurate in HIV programs in resource-limited settings [14].

### Statistical analysis

Baseline characteristics were summarized using descriptive statistics. Groups were compared using χ^2^ tests for categorical and Mann–Whitney U-tests for continuous variables. Changes over time in levels of ALT, viral load, and transient elastography were compared using Wilcoxon signed-rank tests. Intra-individual changes in Fibroscan measurements of more than 20% were considered significant [15].

Logistic regression models were used to study associations between baseline variables and clinically relevant outcomes (adherence, virologic failure, death). Variables with a *p* value below 0.2 in univariable analyses were included in multivariable logistic regression models, using a forward stepwise method. HBV viral load < 69 IU/mL was considered as viral suppression to allow comparison with previous CHB studies [6, 16], and > 1000 IU/mL was considered as major virologic failure.

SPSS version 23.0 software (SPSS Inc., Chicago, IL, USA) was used to analyze the data. The level of significance was set at *p* < 0.05. Results were reported in accordance with the Strengthening the Reporting of Observational studies in Epidemiology (STROBE) statement guidelines [17].

### Ethics

The study was approved by the National Research Ethics Review Committee (Ref. No.: 3.10/829/07) in Ethiopia and by the Regional Committees for Medical and Health Research Ethics (Ref. No.: 2014/1146) in Norway. The study was conducted in accordance with the Declaration of Helsinki. Written informed consent was obtained from all study subjects. 

## Results

### Patient characteristics

**Fig. 1:**
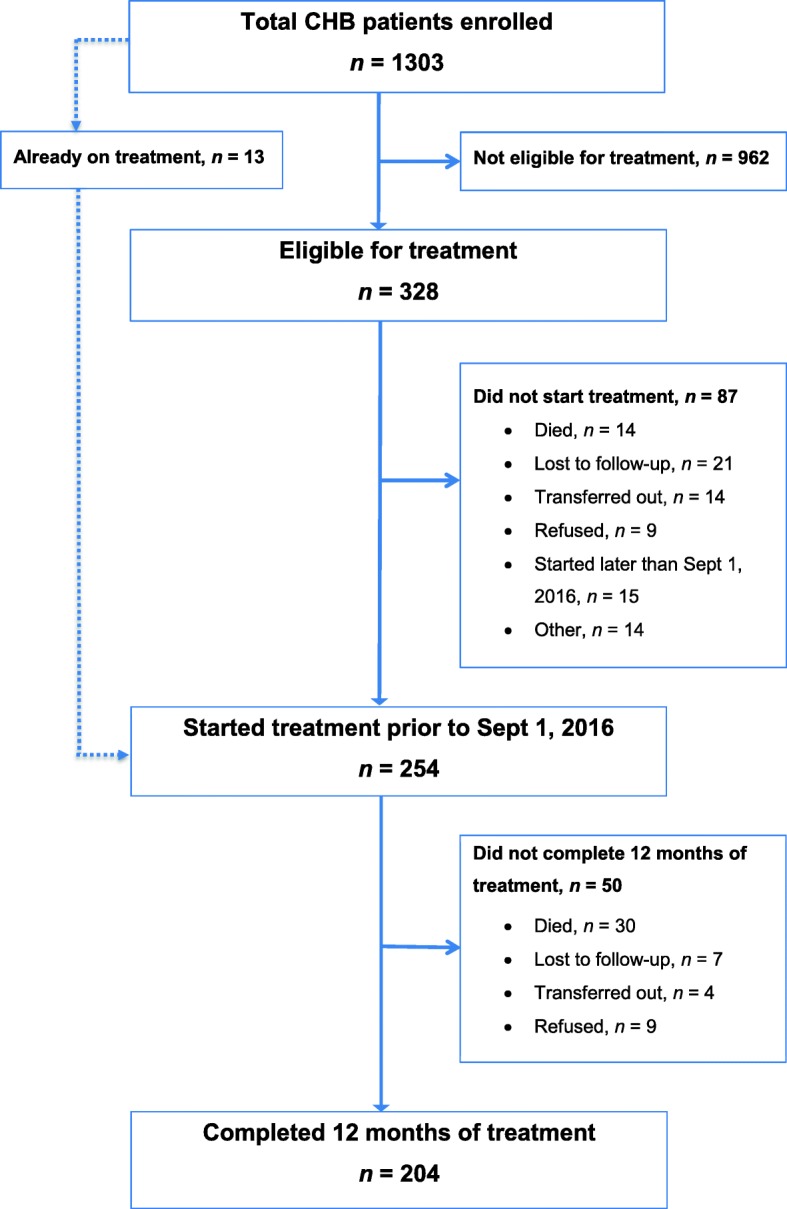
Profile of the hepatitis B treatment program, Addis Ababa, Ethiopia. *CHB* chronic hepatitis B

Between February 9 and December 14, 2015, a total of 1303 adults with CHB were enrolled in the program. Of these, 328 (25.2%) fulfilled the treatment criteria and 254 (19.5%) started treatment prior to September 1, 2016 (Fig. 1). Compared to those who were ineligible for treatment, individuals who met the treatment eligibility criteria were more likely to be men, older, anti-HDV positive, and to have elevated ALT, high viral load and increased liver stiffness (Table 1). Patients eligible for treatment but who did not start it were more likely to have a normal liver stiffness (< 8.0 kPa, 28.6 vs. 10.5%, *p* < 0.001) and a lower APRI score (median 0.31 vs. 0.54, *p* = 0.013) compared to those who started treatment; other distinguishing features could not be identified.

**Table 1:** Baseline characteristics of patients enrolled in a pilot treatment program for chronic hepatitis B, Addis Ababa, Ethiopia

	All patients (*n* = 1303)	Eligible for treatment (*n* = 328)	Not eligible for treatment (*n* = 962)^a^	
	Number (%)	Number (%)	Number (%)	*P*
Men	770 (59.1)	260 (79.3)	500 (52.0)	< 0.001
Age (years)				0.006
18–25	286 (21.9)	64 (19.5)	220 (22.9)	
26–35	549 (42.1)	122 (37.2)	424 (44.1)	
36–45	289 (22.2)	83 (25.3)	202 (21.0)	
> 45	179 (13.7)	59 (18.0)	116 (12.1)	
Marital status				0.667
Single	457 (35.1)	117 (35.7)	338 (35.1)	
Married	796 (61.1)	201 (61.3)	584 (60.7)	
Divorced/widowed	50 (3.8)	10 (3.0)	40 (4.2)	
ALT, U/L^b^				< 0.001
< 40	1014 (78.9)	185 (57.3)	818 (86.2)	
40–79	201 (15.6)	91 (28.2)	108 (11.4)	
≥ 80	70 (5.4)	47 (14.6)	23 (2.4)	
HBV viral load, IU/mL^c^				< 0.001
< 2000	722 (56.5)	107 (33.1)	608 (64.5)	
2000–19,999	256 (20.0)	46 (14.2)	208 (22.1)	
≥ 20,000	301 (23.5)	170 (52.6)	127 (13.5)	
Transient elastography, kPa^d^				< 0.001
< 8.0	879 (74.0)	46 (15.0)	825 (94.5)	
8.0–9.9	67 (5.6)	39 (12.7)	27 (3.1)	
≥ 10.0	242 (20.4)	221 (72.2)	21 (2.4)	
Co-infections				
Anti-HCV positive	28 (2.1)	7 (3.2)	21 (2.6)	0.619
Anti-HDV positive	19 (1.4)	10 (5.9)	9 (1.0)	< 0.001
APRI, median (IQR)^e^	0.24 (0.17–0.36)	0.48 (0.27–0.90)	0.21 (0.16–0.29)	< 0.001
FIB-4, median (IQR)^f^	0.56 (0.41–0.79)	1.06 (0.61–1.91)	0.55 (0.41–0.76)	< 0.001

*ALT* alanine aminotransferase, *HBV* hepatitis B virus, *HCV* hepatitis C virus, *HDV* hepatitis D virus, *APRI* aspartate aminotransferase to platelet ratio index, *IQR* interquartile range

^a^13 patients were already on treatment and could not be assessed for treatment eligibility

^b^18 results missing

^c^ 24 results missing

^d^111 results missing

^e^124 results missing

^f^128 results missing

Among those who started treatment, 197 (77.6%) were men and the median age was 35 years (IQR 27–42). The majority (*n* = 137, 53.9%) were from the capital city, Addis Ababa. The median ALT at baseline was 36 U/L (IQR 24–50), the median viral load was 26,700 IU/mL (IQR 568–9,480,000), and the median Fibroscan value was 15.5 kPa (IQR 9.0–28.9).

### Antiviral treatment

Most patients in this cohort started treatment based on a diagnosis of cirrhosis. A total of 105 (41.3%) patients had clinical ascites or a history of ascites and were classified as decompensated cirrhosis, whereas 81 (31.9%) patients without ascites had a Fibroscan value above 9.9 kPa and were classified as compensated cirrhosis. The remaining started treatment based on significant liver fibrosis (*n* = 30, 11.8%), moderate/severe liver inflammation (*n* = 14, 5.5%), HCC in a first-degree relative (*n* = 3, 1.2%), or other criteria (*n* = 21, 8.3%). The latter groups mainly comprised patients who had initiated treatment through the private sector or ‘black market’, and who could not be assessed using the standard criteria, since most of had suppressed viral load at enrollment.

Among patients who initiated treatment, 111 (43.7%) started immediately (i.e., within 4 weeks of enrollment), mainly due to decompensated cirrhosis, whereas 116 (45.7%) started treatment between 1 and 6 months of enrollment. Only 27 (10.6%) started later than 6 months after enrollment.

### Treatment response

Overall, 30 (11.8 %) patients died within the first 12 months after starting antiviral treatment, 28 of whom had decompensated cirrhosis at baseline. Among the decompensated patients, neither sex (men vs. women; odds ratio (OR) 1.54, 95% confidence interval (CI) 0.40–5.91, *p* = 0.531), age (per 1-year increment; OR 1.01, 95% CI 0.97–1.06, *p* = 0.549), nor co-infections (HCV and/or HDV; OR 1.11, 95% CI 0.20–6.06, *p* = 0.906) predicted subsequent death.

Another 20 (7.9%) patients failed to complete 12 months of TDF therapy; 7 (2.8%) were lost to follow-up, 9 (3.5%) refused to continue treatment for various reasons, and 4 (1.6 %) were transferred out. In the latter group, 3 were diagnosed with HCC and transferred to palliative care, and 1 was diagnosed with HIV on her 3-month follow-up visit and transferred to HIV care. Baseline characteristics of the 3 patients who developed HCC are summarized in Table 2.

**Table 2:** Characteristics of patients who developed hepatocellular carcinoma during the initial 12 months of antiviral treatment

Baseline characteristics	Patient 1	Patient 2	Patient 3
Sex	F	M	M
Age	65	55	50
ALT, U/L	81	30	110
HBV viral load, IU/mL	75	13	10,100
Transient elastography, kPa	29.0	20.6	75.0
Co-infection (HCV/HDV)	No	No	HDV
Co-morbidity	No	IDDM	IDDM
Previous antiviral therapy	No	No	No
Decompensated liver disease	No	Yes	Yes

*ALT* alanine aminotransferase, *HBV* hepatitis B virus, *HCV* hepatitis C virus, *HDV* hepatitis D virus, *IDDM* insulin-dependent diabetes mellitus

**Fig. 2:**
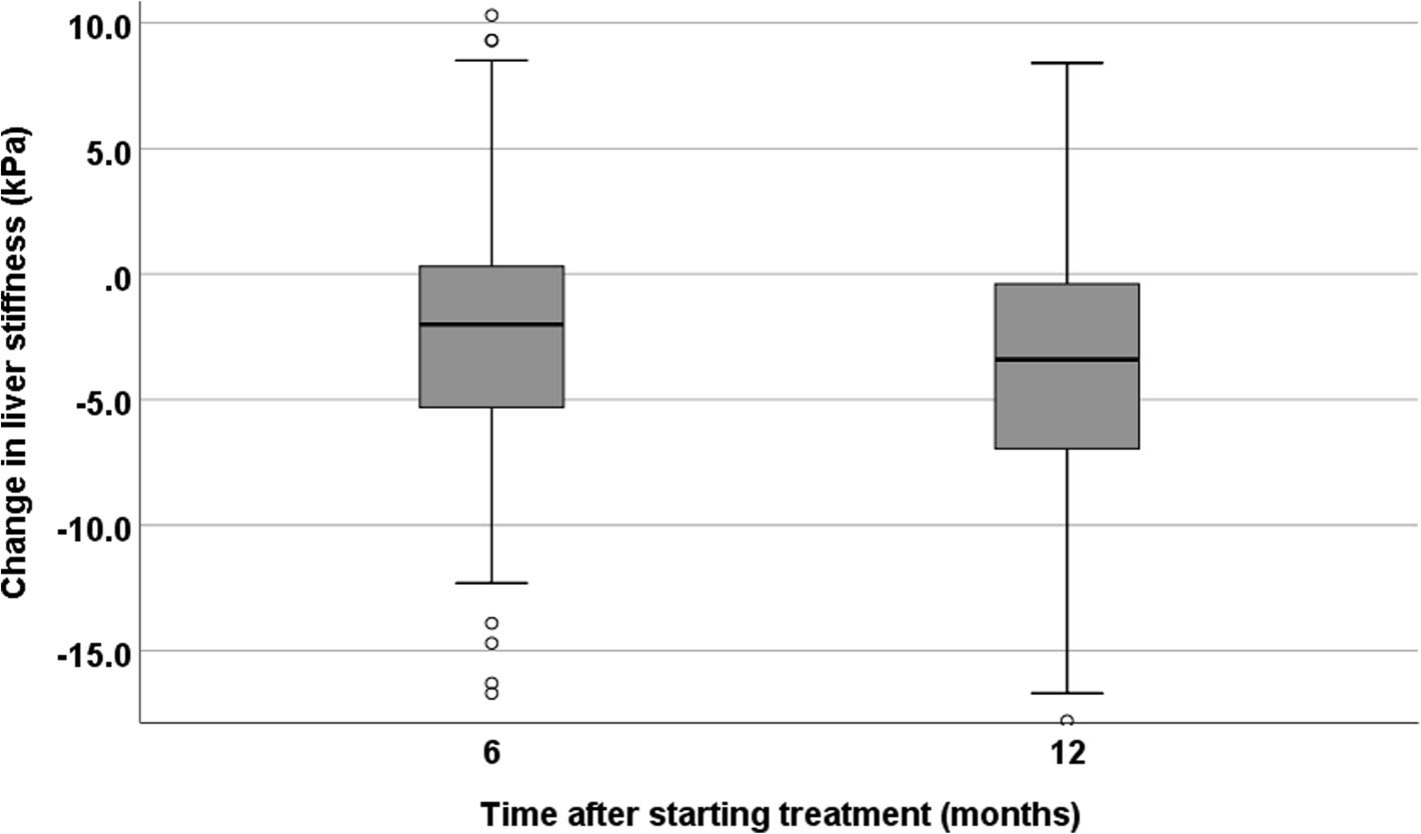
Change in liver stiffness among patients who completed 12 months of hepatitis B treatment

Among the remaining 204 patients who completed 1 year of antiviral treatment, the median ALT was 36 U/L (IQR 24–47) at baseline and 32 U/L (IQR 24–39) at 12 months (*p* = 0.062), and the median Fibroscan value was 12.8 kPa (IQR 8.8–23.6) at baseline and 10.4 kPa (IQR 6.8–17.4) at 12 months (*p* < 0.001). A total of 140 patients had paired Fibroscan results at baseline and 12 months for comparison; the median intra-individual improvement in liver stiffness at 12 months was 3.4 kPa (IQR 0.4–7.0) (Fig. 2). After 12 months of treatment, 83 (59.3%) patients had a significant improvement in liver stiffness, 41 (29.3%) were unchanged (< 20% change from baseline), and 16 (11.4%) deteriorated.

Five individuals (2.5%) experienced HBsAg loss, i.e., they had two consecutively negative HBsAg results; 4 of these developed anti-HBs antibodies. In retrospect, however, 2 cases might have been acute hepatitis B since HBsAg positivity 6 months prior to inclusion could not be documented.

**Fig. 3:**
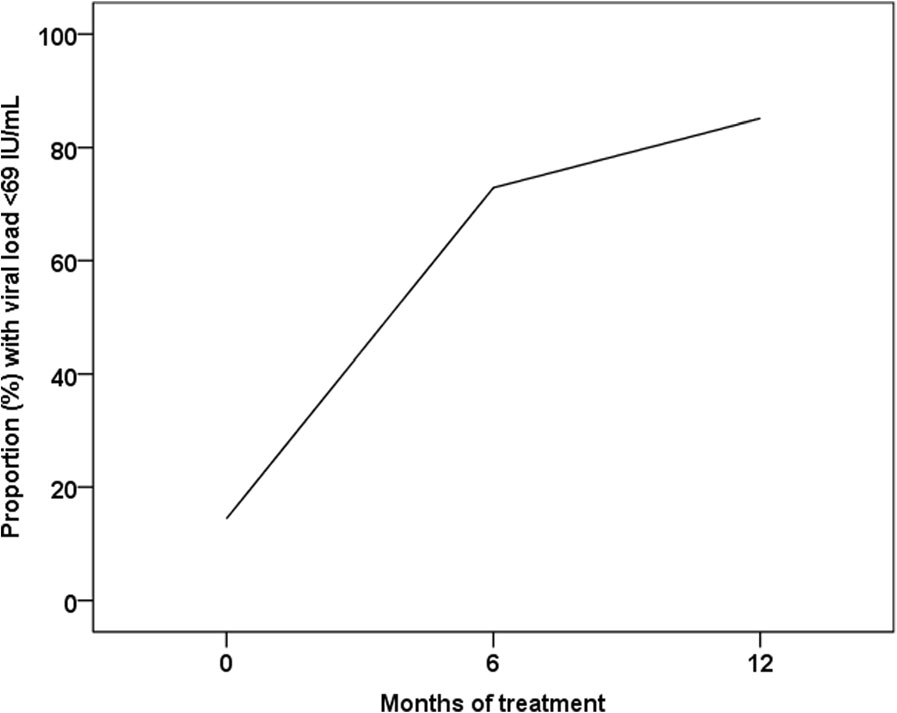
Virologic response to therapy during the first 12 months of antiviral treatment

Out of 189 patients who had a viral load test performed at 12 months, 161 (85.2%) had suppressed viremia (i.e., <69 IU/mL) and only 6 (3.2%) had major virologic failure (i.e., > 1000 IU/mL). Genotypic resistance testing was performed in these 6 samples; 3 had wild type virus and 3 failed amplification due to low viral loads. The proportion of patients with suppressed viremia at 6 and 12 months is given in Fig. 3. Virologic failure (i.e., ≥ 69 IU/mL) at 12 months was associated with high baseline HBV viral load (> 1,000,000 IU/mL) and suboptimal adherence (< 95%) (Table 3).

**Table 3:** Predictors of virologic failure (HBV viral load ≥ 69 IU/mL) after 12 months of antiviral treatment, Addis Ababa, Ethiopia

Baseline variable	Total (*n* = 189)	Virologic failure (*n* = 28)	Crude		Adjusted	
	*n* (%)	*n* (%)	OR (95% CI)	*p*	OR (95% CI)	*p*
Sex						
Women	44 (23.3)	5 (17.9)	1			
Men	145 (76.7)	23 (82.1)	1.52 (0.54–4.26)	0.426		
Age, years (per 1-year increment)	35 (26–41)^a^	35 (26–41) ^a^	1.00 (0.96–1.04)	0.952		
Decompensated liver disease						
No	126 (66.7)	20 (71.4)	1			
Yes	63 (33.3)	8 (28.6)	0.77 (0.32–1.86)	0.563		
HBV viral load, IU/mL^b^						
≤ 10^6^	116 (62.0)	12 (42.9)	1		1	
> 10^6^	71 (38.0)	16 (57.1)	2.52 (1.11–5.71)	0.026	2.41 (1.04–5.55)	0.039
Adherence^c^						
≥ 95%	160 (85.1)	19 (67.9)	1		1	
< 95%	28 (14.9)	9 (32.1)	3.74 (1.47–9.49)	0.006	3.43 (1.33–8.88)	0.011

*HBV* hepatitis B virus, *OR* odds ratio, *CI* confidence interval

^a^Median (interquartile range)

^b^Two missing values

^c^One missing value

TDF therapy was generally well tolerated and only one individual with underlying comorbidities (diabetes mellitus, hypertension, gout, cardiomyopathy, alcohol abuse) discontinued due to progressive worsening of renal function. This patient was under treatment with several drugs, including diuretics and an angiotensin-converting enzyme inhibitor, and the role of TDF in the development of renal failure remains unclear.

### Adherence

Pharmacy refill data was available for 202 out of 204 patients who completed 12 months of antiviral treatment. Out of these, 172 (85.1%) individuals had excellent adherence, i.e., they took more than 95% of their tablets; 24 (11.9%) had medium adherence, i.e., they took between 80 and 95% of their tablets; and 6 had poor adherence, i.e., they took less than 80% of their tablets. Neither sex (men vs. women; OR 1.67, 95% CI 0.60–4.62, *p* = 0.326), age (per 1-year increment; OR 0.98, 95% CI 0.94–1.02, *p* = 0.221), nor decompensated liver disease (OR 1.11, 95% CI 0.20–6.06, *p* = 0.906) were significantly associated with suboptimal adherence (i.e., < 95%).

## Discussion

This report summarizes 1-year treatment results from the largest published cohort of CHB patients in sub-Saharan Africa. The findings indicate that antiviral treatment can be delivered safely and effectively in a low-income setting like Ethiopia using a simplified approach, similar to the early experiences from HIV treatment programs on the continent. In our own setup, the day-to-day management of patients, including the initial patient interview, liver stiffness measurement, blood testing and patient education, was performed by trained nurses, whereas physicians were involved only in the actual decision to start therapy and in the management of complications. Such task shifting, from physicians to trained nurses, has been successful in HIV programs throughout Africa [18], and may also prove useful in the management of other chronic infections like CHB.

The estimated 1-year mortality among patients who started treatment was 11.8%. The high early mortality likely reflects the absence of treatment options for CHB in the country to date; thus, patients with advanced cirrhosis, who are desperate for treatment, might have been overrepresented in our cohort. Indeed, 28 of 30 patients who died had decompensated cirrhosis at enrollment. On the other hand, approximately three-quarters of patients with decompensated cirrhosis in our study were still alive after 12 months of TDF treatment. These results are in line with a study from Korea [19], where 1-year transplantation-free survival was of 87.1% among 70 patients with decompensated cirrhosis treated with entecavir, suggesting that treatment of this group is safe and beneficial. Clearly, however, a major challenge in the Ethiopian setting is the identification of HBV-infected individuals earlier in the course of their illness, prior to the development of severe complications. This would require better access to HBV testing and screening, and improved access to antiviral therapy throughout the country.

In our study, 85.2% of patients achieved viral suppression 12 months after treatment initiation. Only 6 patients had major virologic failure and no genotypic resistance was discovered. This is comparable to studies from high-income settings. Indeed, in the phase 3 study of TDF supported by Gilead Sciences, viral suppression below 69 IU/mL was achieved in 76% and 93% of HBeAg-positive and HBeAg-negative patients, respectively [16]. Moreover, in a real-life multicenter study of 302 CHB patients from 19 countries in Europe, 68% of HBeAg-positive patients and 90% HBeAg-negative patients treated with TDF had a suppressed viral load after 12 months [20]. Studies from low- and middle-income countries are scarce, but in a retrospective study involving 220 cirrhotic patients from India [21], 91.8% had suppressed viremia after 12 months treatment with TDF. Furthermore, in a large real-life study from 50 sites in China [22], viral suppression after 12 months treatment with entecavir was achieved in 64% of patients with compensated liver disease and 68% with decompensated liver disease. Of note, detectable viremia at 12 months does not necessarily imply treatment failure, since it might take longer than 12 months to achieve full viral suppression in individuals with very high baseline viral loads [20].

Interestingly, liver stiffness (measured by Fibroscan) improved significantly during the initial 12 months of therapy. Reversal of liver fibrosis during antiviral therapy has previously been described by Marcellin et al. [6], who reported a significant improvement in fibrosis scores in repeated liver biopsies after 1 and 5 years of TDF treatment. Whether histological improvement translates to reduced risk of HCC and death has yet to be proven in Africa, but experiences from other settings indicate that antiviral treatment significantly reduces the risk of these complications [5].

The program loss in the present study was relatively low; overall, 6.3% were lost to follow-up or self-stopped treatment. Although the follow-up time in our study was shorter, this drop-out rate was lower than results from a recent multicenter study in Germany involving 33 sites across the country [23], where 75% remained in the study after 36 months of TDF treatment. In the only previous publication of CHB treatment in sub-Saharan Africa [9], there was no program loss after 12 months; however, the numbers were small.

Adherence to therapy was high in this cohort; 85.1% of patients took at least 95% of their medication; this is in line with studies from high-income countries. In a study from USA [24], non-adherence was reported in 10–12% of patients after 4 years of HBV therapy, whereas a study from France found non-adherence among 7% of patients after at least 3 months of CHB treatment [25]. High adherence is a prerequisite to achieve the clinical benefits of therapy and to avoid resistance; however, contrary to first-generation anti-HBV drugs, resistance does not seem to be a significant problem with TDF [26]. Previous studies from HIV programs have found that adherence rates in Africa are at least as good as in North America or Europe [27]; our study suggests that the same appears to be true for CHB treatment.

In the present program, treatment decisions were based on viral load testing and liver stiffness measurements. However, in most countries in sub-Saharan Africa, these tests are practically unavailable. Although the WHO recommends the initiation of treatment based on clinical and laboratory markers such as the APRI, experiences from our cohort shows that this marker fails to detect 90% of those in need of treatment [28]. Transient elastography, on the contrary, has shown excellent agreement with liver biopsy [29], and is non-invasive and easy to use, making it an appealing tool in resource-limited settings. Nevertheless, the retail price of a Fibroscan machine is currently beyond the budget of most low-income settings, for which generic competition or other financial mechanisms to improve access to this technology is an urgent priority.

With regard to viral load measurements, we have previously shown that dried blood spots can be used to reliably quantify HBV DNA [30], which means that samples can be sent to a central facility from smaller centers throughout the country. Additionally, the recent development of point-of-care molecular assays for various pathogens represent a new era of near-patient testing, and GeneXpert (Cepheid, Sunnyvale, CA, USA) is expected to launch their HBV DNA viral load kit later in 2018 (personal communication, Emiliano Leone, Cepheid). Improved access to low-cost, robust, point-of-care diagnostics will be a prerequisite to achieve the WHO goal of treating 80% of eligible persons with CHB within 2030 [3]. However, reaching this goal will also require concerted action from international stakeholders and local ministries of health to establish adequate funding mechanisms, develop local treatment guidelines, and remove legislative barriers to generic antiviral drugs.

Our study had certain limitations. First, although the treatment program was set up at a local public hospital, external financial support was provided to purchase a Fibroscan device and run viral load testing. Nevertheless, the clinic was run exclusively with local staff, most of whom had no previous experience with hepatitis B management, and we believe the setup can be duplicated in other countries in sub-Saharan Africa. Second, since this was the first treatment center for CHB in the country, patients with symptomatic (i.e., decompensated) liver disease were overrepresented. The high early mortality in the program reflects this, and as treatment becomes more accessible mortality can be expected to decline.

## Conclusion

The current pilot study showed that treatment for CHB can be successful in terms of adherence to therapy and retention in care. Furthermore, there was significant improvement in liver stiffness during the initial 12 months of treatment, with the majority of patients achieving viral suppression. However, initial mortality was high, given that many patients presented with advanced cirrhosis. Therefore, earlier detection of HBV infection and timely initiation of treatment – prior to the development of irreversible complications – is needed to reach the WHO goal of eliminating viral hepatitis as a public health threat by 2030. This pilot program can provide valuable information for other African countries aiming to expand access to antiviral treatment of CHB.

## Abbreviations

ALT: alanine aminotransferase
APRI: aspartate aminotransferase to platelet ratio index
AST: aspartate aminotransferase
CHB: chronic hepatitis B
CI: confidence interval
EASL: European Association for the Study of the Liver
HBsAg: hepatitis B surface antigen
HBV: hepatitis B virus
HCC: hepatocellular carcinoma
HCV: hepatitis C virus
HDV: hepatitis D virus
HIV: human immunodeficiency virus
IQR: interquartile range
OR: odds ratio
TDF: tenofovir disoproxil fumarate
WHO: World Health Organization
